# Biomarkers for diagnosing serious bacterial infections in older outpatients: a systematic review

**DOI:** 10.1186/s12877-019-1205-0

**Published:** 2019-07-17

**Authors:** Oghenekome A. Gbinigie, Igho J. Onakpoya, Georgia C. Richards, Elizabeth A. Spencer, Constantinos Koshiaris, Niklas Bobrovitz, Carl J. Heneghan

**Affiliations:** Nuffield Department of Primary Care Health Sciences, University of Oxford, Radcliffe Primary Care Building, Radcliffe Observatory Quarter, Woodstock Road, Oxford, OX2 6GG UK

**Keywords:** Biomarkers, Bacterial infections; older adults, Diagnosis, Primary health care

## Abstract

**Background:**

The value of biomarkers for diagnosing bacterial infections in older outpatients is uncertain and limited official guidance exists for clinicians in this area. The aim of this review is to critically appraise and evaluate biomarkers for diagnosing bacterial infections in older adults (aged 65 years and above).

**Methods:**

We searched Medline, Embase, Web of Science and the Cochrane Library, from inception to January 2018. We included studies assessing the diagnostic accuracy of blood, urinary, and salivary biomarkers in diagnosing bacterial infections in older adults. The QUADAS-2 tool was used to assess study quality.

**Results:**

We identified 11 eligible studies of moderate quality (11,034 participants) including 51 biomarkers at varying thresholds for diagnosing bacterial infections. An elevated Procalcitonin (≥ 0.2 ng/mL) may help diagnose bacteraemia in older adults [+ve LR range 1.50 to 2.60]. A CRP ≥ 50 mg/L only raises the probability of bacteraemia by 5%. A positive urine dipstick aids diagnosis of UTI (+ve LR range 1.23 to 54.90), and absence helps rule out UTI (−ve LR range 0.06 to 0.46). An elevated white blood cell count is unhelpful in diagnosing intra-abdominal infections (+ve LR range 0.75 to 2.62), but may aid differentiation of bacterial infection from other acute illness (+ve LR range 2.14 to 7.12).

**Conclusions:**

The limited available evidence suggests that many diagnostic tests useful in younger patients, do not help to diagnose bacterial infections in older adults. Further evidence from high quality studies is urgently needed to guide clinical practice. Until then, symptoms and signs remain the mainstay of diagnosis in community based populations.

**Electronic supplementary material:**

The online version of this article (10.1186/s12877-019-1205-0) contains supplementary material, which is available to authorized users.

## Background

Due to the reduced ability with age to mount an adequate response to pathogenic insults [1], older adults are more susceptible to bacterial infections. Urinary tract infection (UTI) and pneumonia are two common causes of emergency hospital admissions in older adults [2] and cost the NHS £316 million and £235 million, respectively [3].

Serious bacterial infections often present atypically in older adults, creating a diagnostic challenge for clinicians. Fever and other symptoms and signs typically associated with bacterial infections in younger patients may be absent in older adults [4].

The value of biomarkers in diagnosing serious infection in older adults remains uncertain. Older patients hospitalised with community acquired pneumonia (CAP) mount a significantly lower C-reactive protein (CRP) response compared to younger patients, even with more severe disease [5]. Asymptomatic bacteriuria is common in older adults; [6] a positive urine culture could lead to over-treatment [7], antimicrobial side effects, and rising levels of antibiotic resistance.

There is limited official guidance to help clinicians decide which biomarkers help diagnose bacterial infections in older adults. The Scottish Intercollegiate Guidelines Network (SIGN) recognises that bacteriuria may be so common in the older population that urine culture “ceases to be a diagnostic test [8].” They advise against the use of urine dipstick testing [9], however, there is limited evidence as to which tests, if any, are useful.

Therefore, the aim of this review was to systematically review published evidence to determine the value of biomarkers (blood, urinary and salivary) for diagnosing serious bacterial infections in older adults in ambulatory care settings.

## Methods

The protocol was registered on PROSPERO (CRD42018084523). We used methods similar to those published in previous systematic reviews [4, 10] and reported the study according to the Preferred Reporting Items for Systematic Reviews and Meta-Analyses (PRISMA, see Additional file 1).

### Search strategy

We searched Medline, Embase and Web of Science and the Cochrane Library, from inception to January 2018 (See Additional file 2 for full search strategy). We searched Google Scholar for internet proceedings and hand-searched the bibliography of relevant systematic reviews and retrieved articles. Five reviewers (OAG, EAS, GCR, IJO and NB) independently determined eligibility with disagreements resolved through discussion.

### Inclusion criteria

We included observational studies measuring the diagnostic accuracy of a biomarker or combination of biomarkers for diagnosing serious bacterial infections, and providing a reference standard for confirming diagnosis. We defined serious bacterial infections as sepsis (including bacteraemia), pneumonia (excluding infective exacerbations of COPD and asthma), UTI, skin and soft tissue infection (including cellulitis), intra-abdominal infection (cholecystitis, appendicitis, diverticulitis and abscesses), bacterial meningitis, bacterial infective endocarditis and active tuberculosis [11]. Studies have shown these infections cause increased morbidity and mortality in older adults [11, 12].

Included studies had to provide sufficient information to enable extraction of data into two by two tables, allowing calculation of diagnostic accuracy measures. Studies needed to contain a minimum of 10 participants. We included adults aged ≥65 years who at the time of study inclusion were symptomatic with undifferentiated illness. Studies including younger participants were included when age-stratified analyses could be performed for older adults. Studies conducted in outpatient settings (including emergency departments, general practice and outpatient clinics) were eligible for inclusion.

### Exclusion criteria

We excluded studies that were conducted in immunosuppressed participants (e.g. active cancer or receiving chemotherapy) and conducted in developing countries. We also excluded studies in which the index test and reference standard were not performed during the illness episode of the participant. We excluded studies that selected patients on the basis that they all shared a particular co-morbidity. Studies with non-human subjects; and systematic reviews, case reports, case series, case control studies and conference abstracts were excluded. Systematic reviews were used as a point of reference. The exclusion are similar those that have been previously published [4, 10].

### Quality assessment

Three reviewers [OAG, GCR and IJO] independently assessed study quality using the Quality Assessment of Diagnostic Accuracy Studies-2 (QUADAS-2) tool [13]. Disagreements were resolved through discussion; where controversy remained, a third reviewer (CJH) arbitrated.

### Data extraction and analysis

Three reviewers (OAG, GCR and IJO) extracted data independently and in duplicate from included studies into two by two tables. Discrepancies were resolved by discussion. If no consensus could be reached a third reviewer (CK) arbitrated.

We used similar data extraction and analysis methods to a previous diagnostic test accuracy review assessing the value of laboratory tests in identifying serious infections in febrile children [14]. We calculated positive and negative likelihood ratios (+ve LR and –ve LR, respectively) with their 95% confidence intervals, and the pre- and post-test probabilities for each biomarker. A continuity correction of 0.5 was added to empty cells, to ensure calculations were possible [15].

We pre-specified meta-analysis when a biomarker was reported by four or more studies for a particular infection [14], but we had insufficient data to do this. Therefore, results are presented in narrative and on dumbbell plots created in Microsoft Excel (Redmond, WA). Within the plots, we present the pre-test probability of infection (prevalence of infection in the study), and the post-test probability given a positive or negative test result.

## Results

**Fig. 1:**
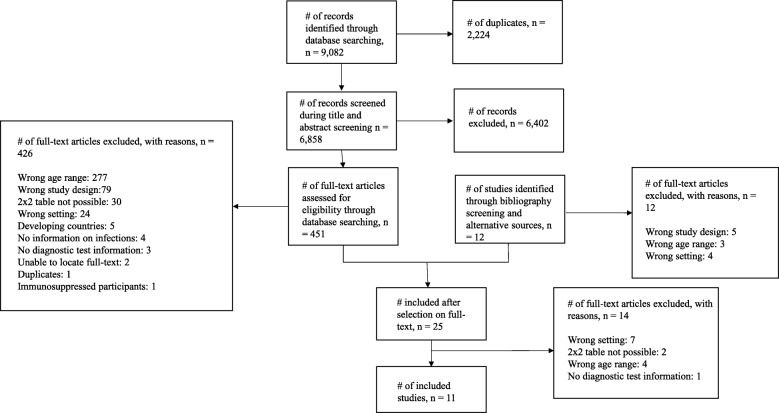
Flow chart showing the process for identification of studies eligible for inclusion

We identified 6,858 non-duplicate results and 463 eligible studies. After full text screening, 11 studies [16–26] met the inclusion criteria (see Fig. 1). Details of these studies are shown in Table 1. Four studies [16, 20, 22, 23] assessed UTI and four [17, 19, 21, 24] assessed bacteraemia. Two studies [18, 26] assessed ‘bacterial infections’ and one [25] assessed intra-abdominal bacterial infections. Eight studies were of prospective diagnostic accuracy design [16–18, 20–22, 24, 26], two were retrospective [19, 25] and one was a prospective cohort study [23]. There were 11,034 participants included in the studies (range 23 to 9,862). Seven studies were conducted in the emergency department (ED) [16, 17, 19, 20, 24–26], and one each in geriatrics clinics [18], a medical centre [21], a day hospital [22] and nursing homes [23]. We did not find any eligible studies assessing the use of salivary biomarkers.

**Table 1:** Characteristics of included studies

Author, year and country	Study Type	Study Setting	Number of participants	Age (Years)	Study Duration	Bacterial Infection(s) investigated	Reference test
Caterino et al. (2015), USA [16]	Diagnostic accuracy study	ED	23	65 years and above	N/A	UTI	Urine culture
Caterino et al. (2004), USA [17]	Diagnostic accuracy study	ED	108	65–100	N/A	Bacteraemia	Blood cultures
Cengiz et al. (2013), Turkey [18]	Diagnostic accuracy study	Geriatrics clinics	110	65–96	N/A	Bacterial Infections	FBC, biochemical parameters, CRP, serum protein electrophoresis, urine examination and Chest X-ray. Further tests (e.g. blood/urine/sputum/wound cultures, abdominal USS, chest and/or abdominal CT, MRI) performed as needed.
Chou et al. (2016), Taiwan [19]	Retrospective observational study of diagnostic accuracy	ED	9862	65 years and above	N/A	Bacteraemia	Blood cultures
Ducharme et al., (2007), Canada [20]	Prospective observational study of diagnostic accuracy	ED	100	65 years and above	N/A	UTI	Urine culture
Dwolatzky et al. (2005), Israel [21]	Prospective observational study of diagnostic accuracy	Medical Centre	80	65–101	N/A	Bacteraemia	Blood cultures
Evans et al. (1991), United Kingdom [22]	Diagnostic accuracy study	Day Hospital	50	65–93	N/A	UTI	Mid-stream urine
Juthani-Mehta et al. (2007), USA [23]	Cohort study	Five nursing homes	101	65 years and above	1 year	UTI	Positive urine culture and greater than 10 white blood cells/mm^3^ on urinalysis
Lai et al. (2010), Taiwan [24]	Diagnostic accuracy study	ED	262	65 years and above	N/A	Bacteraemia	Clinical infection and a positive blood culture
Potts et al. (1999), USA [25]	Retrospective observational study of diagnostic accuracy	ED	117	80 years and above	N/A	Cholecystitis, appendicitis and abscess	Surgical diagnosis
Wasserman et al. (1989), USA [26]	Diagnostic accuracy study	ED	221	70–99	N/A	Bacterial infections	1) Positive blood culture 2) Positive urine culture and pyuria 3) Positive sputum culture and infiltrate on Chest X-ray 4) Positive culture from a normally sterile site or from an abscess cavity.

*Abbreviations*: *CRP* C-reactive protein, *CT* Computerised tomography, *ESR* Erythrocyte sedimentation rate, *MRI* Magnetic Resonance Imaging, *N/A* Not applicable, *USS* Ultrasound Scan, *UTI* Urinary Tract Infection

### Risk of Bias

**Fig. 2:**
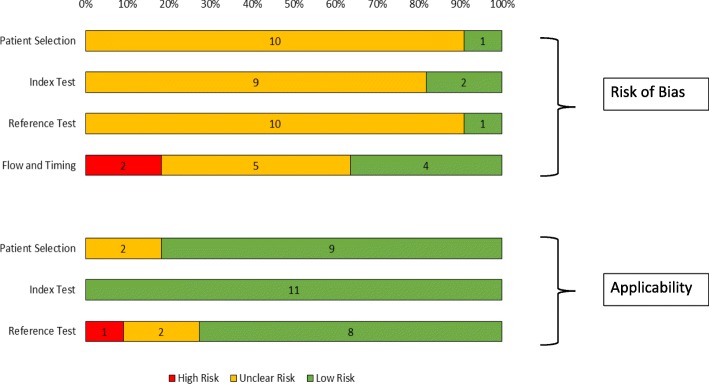
Risk of bias graph. Legend QUADAS-2 Risk of bias and applicability graph showing review authors’ judgements about each domain

**Fig. 3:**
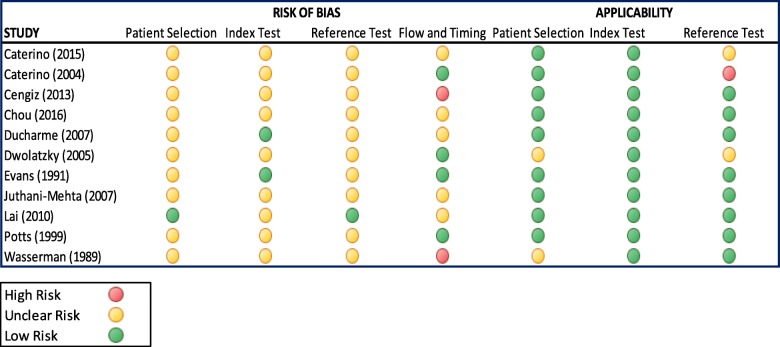
Risk of bias summary. Legend - QUADAS-2 Risk of bias and applicability summary showing review authors’ judgements about each domain

Overall, the 11 studies were of moderate quality (See Figs. 2 and 3). All had good applicability of the index test. Most studies had an unclear risk of bias due to lack of documentation of whether the index test had been interpreted without knowledge of the result of the reference standard, and vice versa.

### Biomarkers

**Fig. 4:**
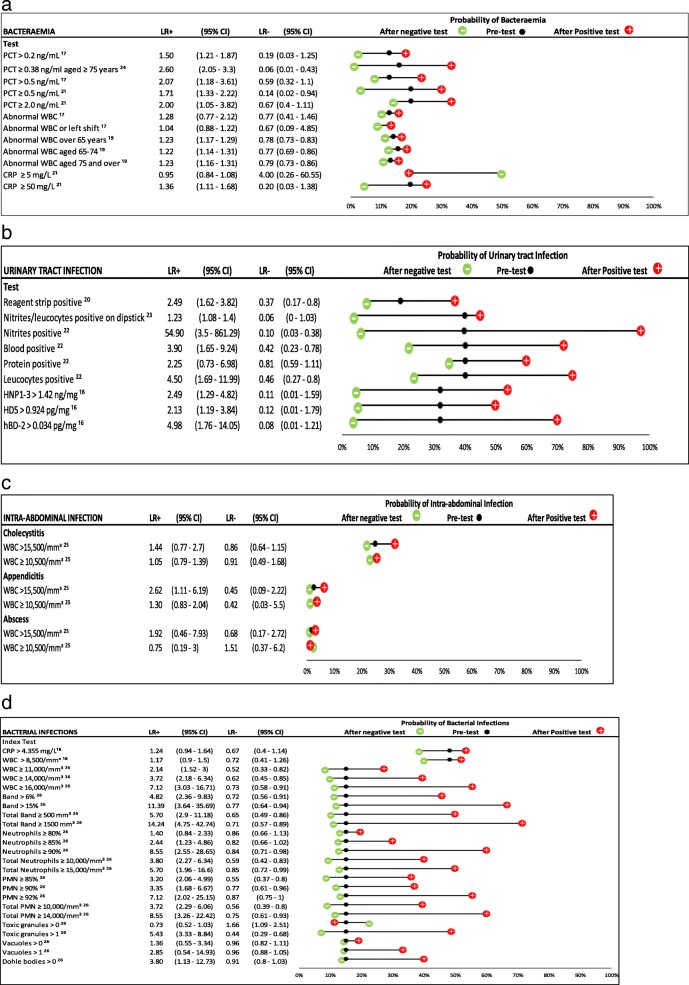
Likelihood ratios and probability plot for diagnostic tests in diagnosing different bacterial infections. Legend – **a**–**d** show likelihood ratios and pre- and post-test probabilities for diagnostic tests in diagnosing different bacterial infections. When possible, age-specific estimates have been given. When not specified, the result applies to patients aged 65 years and above. The figures have been separated according to the type of bacterial infection under investigation. Positive and negative likelihood ratios with 95% confidence intervals are presented for each test. The black dot within the dumbbell plot represents the pre-test probability of infection (i.e. disease prevalence). The red dot represents the probability of the infection after a positive test, and the green dot represents the probability of infection after a negative test. **c** – Estimates derived from people aged 80 years and above. **d** – CRP > 4.355 mg/L and WBC > 8,500/mm^3^ derived from people aged 65 years and above. All other estimates derived from people aged 70 years and above

We calculated the diagnostic accuracy of 51 biomarkers. Figures 4a-d show the likelihood ratios and pre- and post-test probabilities of these biomarkers, categorized by bacterial infection. Two-by-two table data is presented in Additional file 3.

### Bacteraemia

There were five estimates for procalcitonin (PCT) across three studies [17, 21, 24]. Varying cut-off thresholds were used, ranging from a PCT of > 0.2 ng/mL to ≥2.0 ng/mL. Figure 4a shows that having an elevated PCT (> 0.2 ng/mL) helped to diagnose bacteraemia in older adults [+ve LR range 1.50 to 2.60] and raised the probability of bacteraemia by between 5% (PCT > 0.2 ng/mL) [17] and 17% (PCT > 0.38 ng/mL) [24]. However, only two of five estimates found that PCT was a useful rule-out test [21, 24]. Abnormal white blood cell (WBC) count were generally modest [19] to unhelpful [17] for ruling in or out bacteraemia (Fig. 4a). There were two estimates of CRP levels in relation to bacteraemia [21]. A CRP ≥ 50 mg/L, helped to diagnose bacteraemia but only raised the probability of bacteraemia by 5% (Fig. 4a). Having a CRP ≥ 5 mg/L reduced the probability of bacteraemia by 1%.

### Urinary tract infection (UTI)

A urine dipstick positive for nitrites or leucocytes (or in combination), as well as blood, were helpful in making a diagnosis of UTI (+ve LR range 1.23 to 54.90). Four of the five estimates found that absence of these findings on dipstick was a helpful rule out test (−ve LR range 0.06 to 0.46). One study effect size was large [22] and the confidence interval was wide [+ve LR 54.90 (95% CI 3.5 to 861.29)] due to the small number of false positives and relatively small sample size; this result should be interpreted with caution. Protein on urine dipstick was not helpful [22].

One small pilot study (*n* = 23) [16] assessed urinary anti-microbial peptides for diagnosing UTI and suggested they may be useful (See Fig. 4b).

### Intra-abdominal infection

One study [25], published in 1999, assessed the association between WBC count and surgical intra-abdominal infection. Figure 4c shows that a WBC > 15.5 cells/mm^3^ led to a + ve LR 2.62 (95% CI 1.11–6.19), but only increased the probability of appendicitis by 3%. An elevated WBC count was not helpful in diagnosing cholecystitis or intra-abdominal abscess, nor was a normal WBC count useful in ruling out intra-abdominal infections (Fig. 4c).

### Bacterial infections

Two studies [18, 26] assessed the utility of blood tests in diagnosing multiple infections grouped together, mostly of bacterial aetiology (including Pneumonia, UTI, sepsis and bacteraemia). One study [18] assessed patients in a geriatrics clinic with an elevated ESR ≥ 80 mm/h and found that a CRP > 4.36 mg/L or a leucocyte count > 8,500/mm^3^ was not helpful in diagnosing bacterial infections (see Fig. 4d). The other study [26] assessed full blood count and peripheral blood smear for diagnosing bacterial infections in older adults in the ED. They found an elevated WBC count, elevated bands, elevated neutrophils, elevated polymorphonuclear leucocytes (PMNs) and the presence of Dӧhle bodies were all generally helpful in diagnosing and ruling out bacterial infection. These tests raised the probability of bacterial infection by up to 56% [26] (see Fig. 4d).

## Discussion

### Summary

The results of our review were limited by many of the studies having small sample sizes apart from one, the moderate quality, and the limitation to mainly emergency departments. The results do suggest though that an elevated PCT may be a useful test for bacteraemia in primary care, but an abnormal WBC count and elevated CRP are less helpful. A positive urine dipstick may help to diagnose UTI, and there is some evidence that urinary anti-microbial peptides may be helpful. Whereas an elevated WBC count may be helpful in diagnosing appendicitis, it is not of use in diagnosing cholecystitis or intra-abdominal abscess, nor is a normal WBC a useful rule-out test for intra-abdominal infection.

### Comparison with existing literature

Our findings are similar to a previous systematic review by Lee et al [27] that assessed the utility of PCT in diagnosing sepsis in older adults. They included four studies, two of which [16, 17], are included in this review and two further studies [28, 29] that were conducted in the inpatient setting. Overall, they found that PCT may have some value in diagnosing bacterial sepsis [+ve LR 4.77, 95% CI: 2.49–9.13], but not as a standalone test [27]. It is important to note that PCT levels can be affected by co-morbid conditions such as chronic kidney disease [30]. Two included studies [17, 24] excluded certain participants (patients consuming antibiotics in the preceding 48 h, and pre-existing thyroid disease, respectively) to try to minimise this confounding.

A further systematic review [4] assessing clinical predictors of UTI in older adults, haematuria was found to help to diagnose UTI in men, but not in women. This was felt to be related to high levels of atrophic vaginitis in older women [31], which may lead to cross-contamination of urine dipsticks. In the study by Evans et al [22], in which blood on urine dipstick was helpful in diagnosing a UTI, the majority of participants were women (76%). However, our review includes only one estimate of the utility of blood on urine dipstick. Further studies assessing this test are needed to confirm or refute this finding.

### Comparison with existing guidelines

SIGN guidelines for UTI in older adults advise against the use of the urine dipstick as a diagnostic test [9] due to lack of high quality evidence. This advice is echoed by Public Health England guidance [32], because positive urine dipsticks are often be caused by asymptomatic bacteriuria. The results of our review contradict these recommendations, and suggest that a positive urine dipstick for nitrites and leucocytes is helpful in diagnosing UTI in symptomatic older adults. Further studies in this area are required to corroborate or refute these findings.

### Strengths and limitations

To our knowledge, this is the first systematic review assessing the utility of biomarkers in diagnosing bacterial infections in older adult outpatients. Our search strategy was broad so that we could capture as many relevant results as possible. We included studies that were published in all languages and translated eligible non-English language studies.

However, due to the breadth of this review, it is likely that we have missed some relevant studies, particularly unpublished studies. Most of the studies had small sample sizes, and this may have led to spurious results. Due to insufficient numbers of included studies and heterogeneity in the thresholds used as cut-offs to determine test positivity, we were unable to pool the results.

One study [18] included infections that did not fall within our inclusion criteria (e.g. septic arthritis). However, Pneumonia, UTI and sepsis accounted for 85% of the infections assessed, and we therefore believe that the findings are relevant to this review.

We do not have a ‘perfect’ gold standard for diagnosing UTI in older adults; high levels of asymptomatic bacteriuria in older adults means that a positive urine culture does not always represent a UTI. We tried to mitigate this issue by including studies of UTI in symptomatic participants. However, it is possible that a positive urine dipstick result correlates with a positive MSU rather than with the diagnosis of a true UTI.

We are also aware that statistical significance of a test on the basis of a likelihood ratio may not be clinically significant. It is, therefore, useful to interpret likelihood ratios in the context of prior probability using the probability plots.

Incorporation bias is largely unavoidable in studies of diagnostic tests, as clinicians often make diagnoses through combining the results of more than one test and clinical assessment. A single test combined with other information may help to make a diagnosis, but is unlikely to rule in or out a diagnosis in isolation.

It is also possible for confounding to arise through a patient having two bacterial infections, such as bacteraemia and an intra-abdominal infection. The index test might therefore be elevated due to either, or both, of the infections.

### Implications for future research

Few of the studies in this review assessed the utility of combinations of tests; this would be useful in future studies and would allow the development of clinical prediction tools that could be used by clinicians. It would be helpful if these studies used the same or similar thresholds for determining test positivity to facilitate pooling of results. We did not find any eligible studies assessing the utility of biomarkers for diagnosing Pneumonia; research to fill this evidence gap is needed. SIGN guidance recognises that the gold standard for diagnosing UTI in older adults (Urine culture) is imperfect due to high levels of asymptomatic bacteriuria in this age group [6, 8]. We therefore need an alternative gold standard for UTI in older adults. The use of urinary anti-microbial peptides may be promising; further research in this area and assessing other novel tests for UTI in older adults is advised.

### Implications for clinical practice

The evidence from this review suggests some blood and urinary biomarkers are helpful for diagnosing bacterial infections in older adults in the community. However, these findings should be interpreted with caution because they come from a limited number of mostly small studies of moderate quality.

## Conclusions

The limited evidence of moderate quality suggests that an elevated PCT may be helpful for diagnosing bacteraemia, a positive urine dipstick may be helpful in diagnosing UTI. Although an elevated WBC count has limited utility in diagnosing intra-abdominal infections, it may have utility, along with elevated WBC differentials, in differentiating bacterial infections from other acute illness. Further studies of high quality are urgently needed in this area.

## Additional files

Additional file 1: PRISMA checklist. (DOCX 26 kb)

Additional file 2: Search strategy. (DOCX 22 kb)

Additional file 3: **Table S1.** Legend Two by two tables with sensitivities, specificities and their 95% confidence intervals. (PPTX 53 kb)

## Data Availability

Not applicable.

## Abbreviations

+ve LR: Positive likelihood ratio
CAP: Community acquired pneumonia
CRP: C-reactive protein
CT: Computerised tomography
ED: Emergency department
ESR: Erythrocyte sedimentation rate
MRI: Magnetic Resonance Imaging
N/A: Not applicable
PCT: Procalcitonin
PMN: Polymorphonuclear leucocyte
PRISMA: Preferred Reporting Items for Systematic Reviews and Meta-Analyses
QUADAS-2: Quality Assessment of Diagnostic Accuracy Studies-2
SIGN: Scottish Intercollegiate Guidelines Network
USS: Ultrasound
UTI: Urinary Tract Infection
-ve LR: Negative likelihood ratio
WBC: White blood cell
