# Supplementary material for: Biomarkers for diagnosing serious bacterial infections in older outpatients: a systematic review
Source: BMC Geriatr. 2019 Jul 17;19:190. doi: 10.1186/s12877-019-1205-0 (PMC6637629; doi:10.1186/s12877-019-1205-0)
Supplement: Supplementary file 2 — Search strategy. (DOCX 22 kb) [file 12877_2019_1205_MOESM2_ESM.docx]

**Additional File 2 – Search Strategy**

**Medline**

| **# ▲** | **Searches** | **Results** |
| --- | --- | --- |
| **1** | **exp Aged/** | **2760566** |
| **2** | **Geriatrics/** | **28525** |
| **3** | **Geriatric Assessment/** | **23166** |
| **4** | **((old or older) adj2 (people or person? or patient? or adult? or resident?)).ti,ab.** | **203321** |
| **5** | **("old age" or elder* or geriatric* or senior? or veteran?).ti,ab.** | **327056** |
| **6** | **1 or 2 or 3 or 4 or 5** | **2981412** |
| **7** | **Bacterial Infections/** | **66150** |
| **8** | **exp Sepsis/** | **109540** |
| **9** | **Soft Tissue Infections/** | **3075** |
| **10** | **exp Skin Diseases, Infectious/** | **109044** |
| **11** | **exp Staphylococcal Skin Infections/** | **5127** |
| **12** | **Pneumonia, Bacterial/** | **10190** |
| **13** | **Pneumonia/** | **43460** |
| **14** | **Respiratory Tract Infections/** | **35338** |
| **15** | **exp Bronchitis/ or exp Pleurisy/ or Empyema, Pleural/** | **36859** |
| **16** | **tuberculosis/ or tuberculosis, pulmonary/** | **146295** |
| **17** | **exp Intraabdominal Infections/** | **61953** |
| **18** | **exp Urinary Tract Infections/** | **43000** |
| **19** | **Pyelonephritis/** | **13742** |
| **20** | **exp Meningitis, Bacterial/** | **23315** |
| **21** | **endocarditis/ or exp endocarditis, bacterial/** | **26373** |
| **22** | **((bacterial or microbial) adj2 infection?).ti.** | **7062** |
| **23** | **bacter?emi*.ti.** | **11529** |
| **24** | **(sepsis or septic or septic?emi*).ti.** | **48427** |
| **25** | **((systemic or blood*) and infection?).ti.** | **12271** |
| **26** | **((serious or sever*) and infection?).ti.** | **7724** |
| **27** | **common infection?.ti.** | **207** |
| **28** | **((skin adj2 infection?) or cellulitis).ti.** | **4585** |
| **29** | **(((respiratory or chest or lung) adj2 infection) or pneumonia or bronchitis or bronchiolitis or tuberculosis).ti.** | **187773** |
| **30** | **(((intraabdom* or intra-abdom* or abdom*) adj2 (infection? or abscess*)) or appendicitis or peritonitis or diverticulitis).ti.** | **29809** |
| **31** | **(urin* adj2 infection?).ti.** | **15525** |
| **32** | **(pyelonephritis or ((kidney or renal) adj2 infection?)).ti.** | **8206** |
| **33** | **meningitis.ti.** | **26654** |
| **34** | **endocarditis.ti.** | **19702** |
| **35** | **7 or 8 or 9 or 10 or 11 or 12 or 13 or 14 or 16 or 17 or 18 or 19 or 20 or 21 or 22 or 23 or 24 or 25 or 26 or 27 or 28 or 29 or 30 or 31 or 32 or 33 or 34** | **758849** |
| **36** | **diagnostic tests, routine/** | **9668** |
| **37** | **Biomarkers/** | **220310** |
| **38** | **exp Hematologic Tests/** | **235016** |
| **39** | **Saliva/an [Analysis]** | **4184** |
| **40** | **exp Blood/an [Analysis]** | **23677** |
| **41** | **Urine/an [Analysis]** | **2820** |
| **42** | **cytokines/ or exp interleukins/** | **315500** |
| **43** | **C-Reactive Protein/** | **39593** |
| **44** | **Leukocyte L1 Antigen Complex/** | **1828** |
| **45** | **Haptoglobins/** | **6126** |
| **46** | **Calcitonin/** | **15138** |
| **47** | **Tumor Necrosis Factor-alpha/** | **112815** |
| **48** | **Platelet Factor 4/** | **2500** |
| **49** | **exp Matrix Metalloproteinases/** | **40899** |
| **50** | **exp Blood Cell Count/** | **132905** |
| **51** | **exp Blood Cells/** | **896844** |
| **52** | **Protein Binding/ and Heparin/** | **2260** |
| **53** | **Liver Function Tests/** | **27386** |
| **54** | **exp Kidney Function Tests/** | **72207** |
| **55** | **Blood Glucose/** | **148773** |
| **56** | **Lactic Acid/** | **38801** |
| **57** | **Electrolytes/** | **24396** |
| **58** | **marker?.ti. or (((biological or disease or infection) adj5 marker?) or biomarker?).ti,ab.** | **301652** |
| **59** | **((blood or hematolog* or haematolog* or plasma or serum) adj3 (test* or diagnos*)).ti,ab.** | **90695** |
| **60** | **(saliva* adj3 (test* or diagnos*)).ti,ab.** | **3797** |
| **61** | **(urin* adj3 (test* or diagnos*)).ti,ab.** | **16556** |
| **62** | **(interleukin* or interferon* or cytokine* or c-reactive protein or erythrocyte sedimentation rate or blood sedimentation rate or calprotectin or haptoblobin? or calcitonin or procalcitonin or prostaglandin? or tumo?r necrosis factor or platelet factor or pf-4 or matrix metalloproteinase or leukocyte? or neutrophil? or lymphocyte? or eosinophil? or basophil? or presepsin).ti,ab.** | **1164461** |
| **63** | **(heparin binding protein? or ((liver or hepat*) adj2 test*)).ti,ab.** | **20635** |
| **64** | **((kidney or renal) adj2 test*).ti,ab.** | **5677** |
| **65** | **((blood or serum) adj2 glucose).ti,ab. or glucose.ti.** | **162098** |
| **66** | **lactate.ti,ab.** | **91845** |
| **67** | **electrolyte?.ti,ab.** | **62973** |
| **68** | **36 or 37 or 38 or 39 or 40 or 41 or 42 or 43 or 44 or 45 or 46 or 47 or 48 or 49 or 50 or 51 or 52 or 53 or 54 or 55 or 56 or 57 or 58 or 59 or 60 or 61 or 62 or 63 or 64 or 65 or 66 or 67** | **2773981** |
| **69** | **exp Point-of-Care Systems/** | **10862** |
| **70** | **Ambulatory Care/** | **39769** |
| **71** | **ambulatory care facilities/ or community health centers/** | **23094** |
| **72** | **general practice/ or family practice/** | **71478** |
| **73** | **Office Visits/** | **6345** |
| **74** | **Primary Health Care/** | **66199** |
| **75** | **general practitioners/ or physicians, family/ or physicians, primary care/** | **23940** |
| **76** | **Emergency Service, Hospital/ or Emergency Medical Services/** | **92029** |
| **77** | **((("point of care" or poc or rapid or "near patient" or bedside or bed-side) adj3 (test* or diagnos*)) or poct).ti,ab.** | **39101** |
| **78** | **(ambulatory adj5 (department? or dept* or ward? or room? or unit? or service? or care or setting? or facilit*)).ti,ab.** | **16879** |
| **79** | **((general or family) adj2 (practi* or physician? or doctor?)).ti,ab.** | **107424** |
| **80** | **((primary* adj3 (care or health*)) or community or communities or population).ti,ab.** | **1632594** |
| **81** | **(clinic? or office or visit? or health centre? or health center? or medical centre? or medical center?).ti,ab.** | **538222** |
| **82** | **("out of hours" or ooh or "after hours").ti,ab.** | **3925** |
| **83** | **(emergency adj5 (department? or dept* or ward? or room? or unit? or service? or care or setting? or facilit*)).ti,ab.** | **115729** |
| **84** | **residential facilities/ or assisted living facilities/ or homes for the aged/ or exp nursing homes/** | **46068** |
| **85** | **Institutionalization/ or Long-Term Care/** | **28777** |
| **86** | **((nursing or residential or longterm or long-term or institutional) adj2 home?).ti,ab.** | **30693** |
| **87** | **((residential or longterm or long-term) adj (care or facilit*)).ti,ab.** | **21489** |
| **88** | **((care adj2 home?) or homecare).ti,ab.** | **25123** |
| **89** | **exp Home Care Services/** | **43882** |
| **90** | **((home or domiciliary) adj (visit* or call*)).ti,ab.** | **7536** |
| **91** | **69 or 70 or 71 or 72 or 73 or 74 or 75 or 76 or 77 or 78 or 79 or 80 or 81 or 82 or 83 or 84 or 85 or 86 or 87 or 88 or 89 or 90** | **2440903** |
| **92** | **6 and 35 and 68 and 91** | **3435** |

**Embase**

| **# ▲** | **Searches** | **Results** |
| --- | --- | --- |
| **1** | **exp aged/** | **2662393** |
| **2** | **geriatrics/ or exp elderly care/** | **101931** |
| **3** | **geriatric assessment/** | **13089** |
| **4** | **((old or older) adj2 (people or person? or patient? or adult? or resident?)).ti,ab.** | **276991** |
| **5** | **("old age" or elder* or geriatric* or senior? or veteran?).ti,ab.** | **448249** |
| **6** | **1 or 2 or 3 or 4 or 5** | **2984659** |
| **7** | ***Bacterial Infection/** | **59094** |
| **8** | **exp *Sepsis/** | **88875** |
| **9** | ***Soft Tissue Infection/** | **3145** |
| **10** | **exp *Skin Diseases, Infectious/** | **101084** |
| **11** | **exp *skin infection/** | **101084** |
| **12** | **exp *bacterial pneumonia/** | **14295** |
| **13** | ***Pneumonia/** | **43830** |
| **14** | ***Respiratory Tract Infection/** | **25404** |
| **15** | **exp *lower respiratory tract infection/** | **120453** |
| **16** | ***lung tuberculosis/ or *tuberculosis/** | **113592** |
| **17** | **exp *abdominal Infection/** | **7843** |
| **18** | **exp *Urinary Tract Infection/** | **39202** |
| **19** | **exp *Pyelonephritis/** | **11807** |
| **20** | ***meningitis/ or *bacterial meningitis/ or *group b streptococcal meningitis/ or exp *pneumococcal meningitis/** | **26387** |
| **21** | ***endocarditis/ or exp *bacterial endocarditis/** | **24582** |
| **22** | **((bacterial or microbial) adj2 infection?).ti.** | **8763** |
| **23** | **bacter?emi*.ti.** | **13429** |
| **24** | **(sepsis or septic or septic?emi*).ti.** | **62490** |
| **25** | **((systemic or blood*) and infection?).ti.** | **15328** |
| **26** | **((serious or sever*) and infection?).ti.** | **9937** |
| **27** | **common infection?.ti.** | **217** |
| **28** | **((skin adj2 infection?) or cellulitis).ti.** | **5567** |
| **29** | **(((respiratory or chest or lung) adj2 infection) or pneumonia or bronchitis or bronchiolitis or tuberculosis).ti.** | **190956** |
| **30** | **(((intraabdom* or intra-abdom* or abdom*) adj2 (infection? or abscess*)) or appendicitis or peritonitis or diverticulitis).ti.** | **31979** |
| **31** | **(urin* adj2 infection?).ti.** | **21402** |
| **32** | **(pyelonephritis or ((kidney or renal) adj2 infection?)).ti.** | **9176** |
| **33** | **meningitis.ti.** | **26559** |
| **34** | **endocarditis.ti.** | **21932** |
| **35** | **7 or 8 or 9 or 10 or 11 or 12 or 13 or 14 or 16 or 17 or 18 or 19 or 20 or 21 or 22 or 23 or 24 or 25 or 26 or 27 or 28 or 29 or 30 or 31 or 32 or 33 or 34** | **682554** |
| **36** | ***diagnostic test/** | **10477** |
| **37** | **Biomarker/** | **205262** |
| **38** | **exp blood examination/** | **230936** |
| **39** | **saliva analysis/** | **7478** |
| **40** | **exp urinalysis/** | **92116** |
| **41** | **exp cytokine/** | **1269611** |
| **42** | **C Reactive Protein/** | **137378** |
| **43** | **leukocyte antigen/** | **12437** |
| **44** | **Haptoglobin/** | **11436** |
| **45** | **procalcitonin/** | **8796** |
| **46** | **Tumor Necrosis Factor/** | **70177** |
| **47** | **exp Matrix Metalloproteinases/** | **24521** |
| **48** | **exp Blood Cell Count/** | **271428** |
| **49** | **exp Blood Cell/** | **1333706** |
| **50** | **heparin binding protein/** | **1110** |
| **51** | **Liver Function Test/** | **39160** |
| **52** | **Kidney Function Test/** | **14145** |
| **53** | **glucose blood level/ or glucose tolerance test/** | **235707** |
| **54** | **Lactic Acid/** | **64025** |
| **55** | **Electrolyte/** | **36794** |
| **56** | **marker?.ti. or (((biological or disease or infection) adj5 marker?) or biomarker?).ti,ab.** | **441337** |
| **57** | **((blood or hematolog* or haematolog* or plasma or serum) adj3 (test* or diagnos*)).ti,ab.** | **132829** |
| **58** | **(saliva* adj3 (test* or diagnos*)).ti,ab.** | **4647** |
| **59** | **(urin* adj3 (test* or diagnos*)).ti,ab.** | **23422** |
| **60** | **(interleukin* or interferon* or cytokine* or c-reactive protein or erythrocyte sedimentation rate or blood sedimentation rate or calprotectin or haptoblobin? or calcitonin or procalcitonin or prostaglandin? or tumo?r necrosis factor or platelet factor or pf-4 or matrix metalloproteinase or leukocyte? or neutrophil? or lymphocyte? or eosinophil? or basophil? or presepsin).ti,ab.** | **1497607** |
| **61** | **(heparin binding protein? or ((liver or hepat*) adj2 test*)).ti,ab.** | **30807** |
| **62** | **((kidney or renal) adj2 test*).ti,ab.** | **7562** |
| **63** | **((blood or serum) adj2 glucose).ti,ab. or glucose.ti.** | **214386** |
| **64** | **lactate.ti,ab.** | **113052** |
| **65** | **electrolyte?.ti,ab.** | **69418** |
| **66** | **36 or 37 or 38 or 39 or 40 or 41 or 42 or 43 or 44 or 45 or 46 or 47 or 48 or 49 or 50 or 51 or 52 or 53 or 54 or 55 or 56 or 57 or 58 or 59 or 60 or 61 or 62 or 63 or 64 or 65** | **4098347** |
| **67** | **"point of care testing"/** | **9391** |
| **68** | **Ambulatory Care/** | **34813** |
| **69** | **community care/** | **54643** |
| **70** | **general practice/** | **77361** |
| **71** | **health center/ or out-of-hours care/** | **29109** |
| **72** | **Primary Health Care/** | **57034** |
| **73** | **general practitioner/** | **83925** |
| **74** | **emergency ward/** | **106147** |
| **75** | **((("point of care" or poc or rapid or "near patient" or bedside or bed-side) adj3 (test* or diagnos*)) or poct).ti,ab.** | **51874** |
| **76** | **(ambulatory adj5 (department? or dept* or ward? or room? or unit? or service? or care or setting? or facilit*)).ti,ab.** | **22530** |
| **77** | **((general or family) adj2 (practi* or physician? or doctor?)).ti,ab.** | **133619** |
| **78** | **((primary* adj3 (care or health*)) or community or communities or population).ti,ab.** | **2123071** |
| **79** | **(clinic? or office or visit? or health centre? or health center? or medical centre? or medical center?).ti,ab.** | **803435** |
| **80** | **("out of hours" or ooh or "after hours").ti,ab.** | **5495** |
| **81** | **(emergency adj5 (department? or dept* or ward? or room? or unit? or service? or care or setting? or facilit*)).ti,ab.** | **170342** |
| **82** | **nursing home/ or residential home/ or assisted living facility/ or home for the aged/** | **60770** |
| **83** | **institutional care/ or long term care/** | **117665** |
| **84** | **((nursing or residential or longterm or long-term or institutional) adj2 home?).ti,ab.** | **38233** |
| **85** | **((residential or longterm or long-term) adj (care or facilit*)).ti,ab.** | **26527** |
| **86** | **((care adj2 home?) or homecare).ti,ab.** | **29863** |
| **87** | **home care/** | **55887** |
| **88** | **((home or domiciliary) adj (visit* or call*)).ti,ab.** | **9554** |
| **89** | **67 or 68 or 69 or 70 or 71 or 72 or 73 or 74 or 75 or 76 or 77 or 78 or 79 or 80 or 81 or 82 or 83 or 84 or 85 or 86 or 87 or 88** | **3284548** |
| **90** | **6 and 35 and 66 and 89** | **4998** |

**Web of Knowledge**

| **Set** | **Results** | **Save search history and/or create an alertOpen a saved search history** |
| --- | --- | --- |
| **# 5** | **628** | **#4 AND #3 AND #2 AND #1** |
| **# 4** | **2,619,364** | **TOPIC: ((("point of care" or poc or rapid or "near patient" or bedside or bed-side) NEAR/3 (test* or diagnos*)) or poct) OR TOPIC: ((ambulatory NEAR/5 (department? or dept* or ward? or room? or unit? or service? or care or setting? or facilit*))) OR TOPIC: ((general or family) NEAR/2 (practi* or physician? or doctor?)) OR TOPIC: ((primary* NEAR/3 (care or health*)) or community or communities or population) OR TOPIC: (clinic? or office or visit? or "health centre?" or "health center?" or "medical centre?" or "medical center?") OR TOPIC: ("out of hours" or ooh or "after hours") OR TOPIC: ((emergency NEAR/5 (department? or dept* or ward? or room? or unit? or service? or care or setting? or facilit*))) OR TOPIC: ((nursing or residential or longterm or long-term or institutional) NEAR/2 home?) OR TOPIC: ((residential or longterm or long-term) NEXT (care or facilit*)) OR TOPIC: ((care NEAR/2 home?) or homecare or "home visit*" OR "home call*" OR "domiciliary visit*" OR "domiciliary call*")** |
| **# 3** | **1,879,248** | **TITLE: (marker* OR glucose) OR TOPIC: (((biological or disease or infection) NEAR/5 marker?) or biomarker?) OR TOPIC: ((blood OR haematolog* OR hematolog* OR plasma OR serum OR saliva OR urin*) NEAR/3 (test* OR diagnos*)) OR TOPIC: (interleukin* or interferon* or cytokine* or "c-reactive protein" or "erythrocyte sedimentation rate" or "blood sedimentation rate" or calprotectin or haptoblobin? or calcitonin or procalcitonin or prostaglandin? or "tumo?r necrosis factor" or "platelet factor" or pf-4 or "matrix metalloproteinase" or leukocyte? or neutrophil? or lymphocyte? or eosinophil? or basophil? or presepsin) OR TOPIC: ("heparin binding protein?" or ((liver or hepat) NEAR/2 test*)) OR TOPIC: (((kidney or renal) NEAR/2 test*)) OR TOPIC: (((blood or serum) NEAR/2 glucose)) OR TOPIC: (lactate OR electrolyte?)** |
| **# 2** | **574,413** | **TS=(((bacterial or microbial) NEAR/2 infection?) OR sepsis OR septic* OR "serious infection*" OR "severe infection*" OR "common infection*") OR TS=("skin infection*" OR cellulitis) OR TS=(((respiratory or chest or lung) NEAR/2 infection) or pneumonia or bronchitis or bronchiolitis or tuberculosis) OR TS=(((intraabdom* or intra-abdom* or abdom*) NEAR/2 (infection? or abscess*)) or appendicitis or peritonitis or diverticulitis) OR TS=((urin* NEAR/2 infection*) OR pyelonephritis or ((kidney or renal) NEAR/2 infection?)) OR TS=(meningitis) OR TS=(endocarditis)** |
| **# 1** | **405,655** | **TOPIC: (((old or older) NEAR/2 (people or person? or patient? or adult? or resident?))) OR TOPIC: ("old age" or elder* or geriatric* or senior? or veteran?)** |

**Cochrane**

| **ID** | **Search** |
| --- | --- |
| **#1** | **MeSH descriptor: [Aged] explode all trees** |
| **#2** | **MeSH descriptor: [Geriatrics] explode all trees** |
| **#3** | **MeSH descriptor: [Geriatric Assessment] explode all trees** |
| **#4** | **((old or older) near/2 (people or person? or patient? or adult? or resident?)):ti,ab,kw and "old age" or elder* or geriatric* or senior? or veteran?:ti,ab,kw (Word variations have been searched)** |
| **#5** | **#1 or #2 or #3 or #4** |
| **#6** | **MeSH descriptor: [Bacterial Infections] explode all trees** |
| **#7** | **MeSH descriptor: [Sepsis] explode all trees** |
| **#8** | **MeSH descriptor: [Soft Tissue Infections] explode all trees** |
| **#9** | **MeSH descriptor: [Skin Diseases, Infectious] explode all trees** |
| **#10** | **MeSH descriptor: [Pneumonia] this term only** |
| **#11** | **MeSH descriptor: [Pneumonia, Bacterial] explode all trees** |
| **#12** | **MeSH descriptor: [Empyema, Pleural] 1 tree(s) exploded** |
| **#13** | **MeSH descriptor: [Bronchitis] explode all trees** |
| **#14** | **MeSH descriptor: [Tuberculosis] this term only** |
| **#15** | **MeSH descriptor: [Tuberculosis, Pulmonary] explode all trees** |
| **#16** | **MeSH descriptor: [Intraabdominal Infections] explode all trees** |
| **#17** | **MeSH descriptor: [Urinary Tract Infections] explode all trees** |
| **#18** | **MeSH descriptor: [Pyelonephritis] explode all trees** |
| **#19** | **MeSH descriptor: [Meningitis, Bacterial] explode all trees** |
| **#20** | **MeSH descriptor: [Endocarditis] explode all trees** |
| **#21** | **((bacterial or microbial) near/2 infection?) or bacteraemi* or bacteremi* or sepsis or septic* or "systemic infection*" or "serious infection*" or "common infection*":ti,ab,kw or "skin infection*" or cellulitis or ((respirat* or chest or lung or pulmonary) near/2 infection*) or pneomonia or bronchitis or bronchiolitis or tuberculosis:ti,ab,kw or ((intraabdom* or intra-abdom* or abdom*) near/2 (infection? or abscess*)) or appendicitis or peritonitis or diverticulitis:ti,ab,kw or (urin* near/2 infection*) or pyelonephritis or ((kidney or renal) near/2 infection?):ti,ab,kw or meningitis or endocarditis:ti,ab,kw (Word variations have been searched)** |
| **#22** | **#6 or #7 or #8 or #9 or #10 or #11 or #12 or #13 or #14 or #15 or #16 or #17 or #18 or #19 or #20 or #21** |
| **#23** | **MeSH descriptor: [Diagnostic Tests, Routine] explode all trees** |
| **#24** | **MeSH descriptor: [Biomarkers] this term only** |
| **#25** | **MeSH descriptor: [Hematologic Tests] explode all trees** |
| **#26** | **MeSH descriptor: [Blood] explode all trees** |
| **#27** | **MeSH descriptor: [Saliva] explode all trees** |
| **#28** | **MeSH descriptor: [Urine] explode all trees** |
| **#29** | **MeSH descriptor: [Cytokines] this term only** |
| **#30** | **MeSH descriptor: [Interleukins] explode all trees** |
| **#31** | **MeSH descriptor: [C-Reactive Protein] explode all trees** |
| **#32** | **MeSH descriptor: [Leukocyte L1 Antigen Complex] this term only** |
| **#33** | **MeSH descriptor: [Haptoglobins] explode all trees** |
| **#34** | **MeSH descriptor: [Calcitonin] explode all trees** |
| **#35** | **MeSH descriptor: [Tumor Necrosis Factor-alpha] explode all trees** |
| **#36** | **MeSH descriptor: [Platelet Factor 4] explode all trees** |
| **#37** | **MeSH descriptor: [Matrix Metalloproteinases] explode all trees** |
| **#38** | **MeSH descriptor: [Blood Cell Count] explode all trees** |
| **#39** | **MeSH descriptor: [Blood Cells] explode all trees** |
| **#40** | **MeSH descriptor: [Liver Function Tests] explode all trees** |
| **#41** | **MeSH descriptor: [Kidney Function Tests] explode all trees** |
| **#42** | **MeSH descriptor: [Blood Glucose] explode all trees** |
| **#43** | **MeSH descriptor: [Lactic Acid] explode all trees** |
| **#44** | **MeSH descriptor: [Electrolytes] explode all trees** |
| **#45** | **marker* or glucose:ti or ((biological or disease or infection) near marker?) or biomarker*:ti,ab,kw or ((blood or hematolog* or haematolog* or plasma or serum or saliva* or urin*) near/3 (test* or diagnos*)):ti,ab,kw or interleukin* or interferon* or cytokine* or "c-reactive protein" or "erythrocyte sedimentation rate" or "blood sedimentation rate" or calprotectin or haptoblobin? or calcitonin or procalcitonin or prostaglandin? or "tumo?r necrosis factor" or "platelet factor" or pf-4 or "matrix metalloproteinase" or leukocyte? or neutrophil? or lymphocyte? or eosinophil? or basophil? or presepsin:ti,ab,kw or "heparin binding protein*" or ((liver or hepat* or kidney* or renal) near/2 test*) or "blood glucose" or lactate or electrolyte*:ti,ab,kw (Word variations have been searched)** |
| **#46** | **#23 or #24 or #25 or #26 or #27 or #28 or #29 or #30 or #31 or #32 or #33 or #34 or #35 or #36 or #37 or #38 or #39 or #40 or #41 or #42 or #43 or #44 or #45** |
| **#47** | **#5 and #22 and #46** |
