# Supplementary material for: Biomarkers for diagnosing serious bacterial infections in older outpatients: a systematic review
Source: BMC Geriatr. 2019 Jul 17;19:190. doi: 10.1186/s12877-019-1205-0 (PMC6637629; doi:10.1186/s12877-019-1205-0)
Supplement: Supplementary file 3 — Table S1. Legend Two by two tables with sensitivities, specificities and their 95% confidence intervals. (PPTX 53 kb) [file 12877_2019_1205_MOESM3_ESM.pptx]

## Slide 1
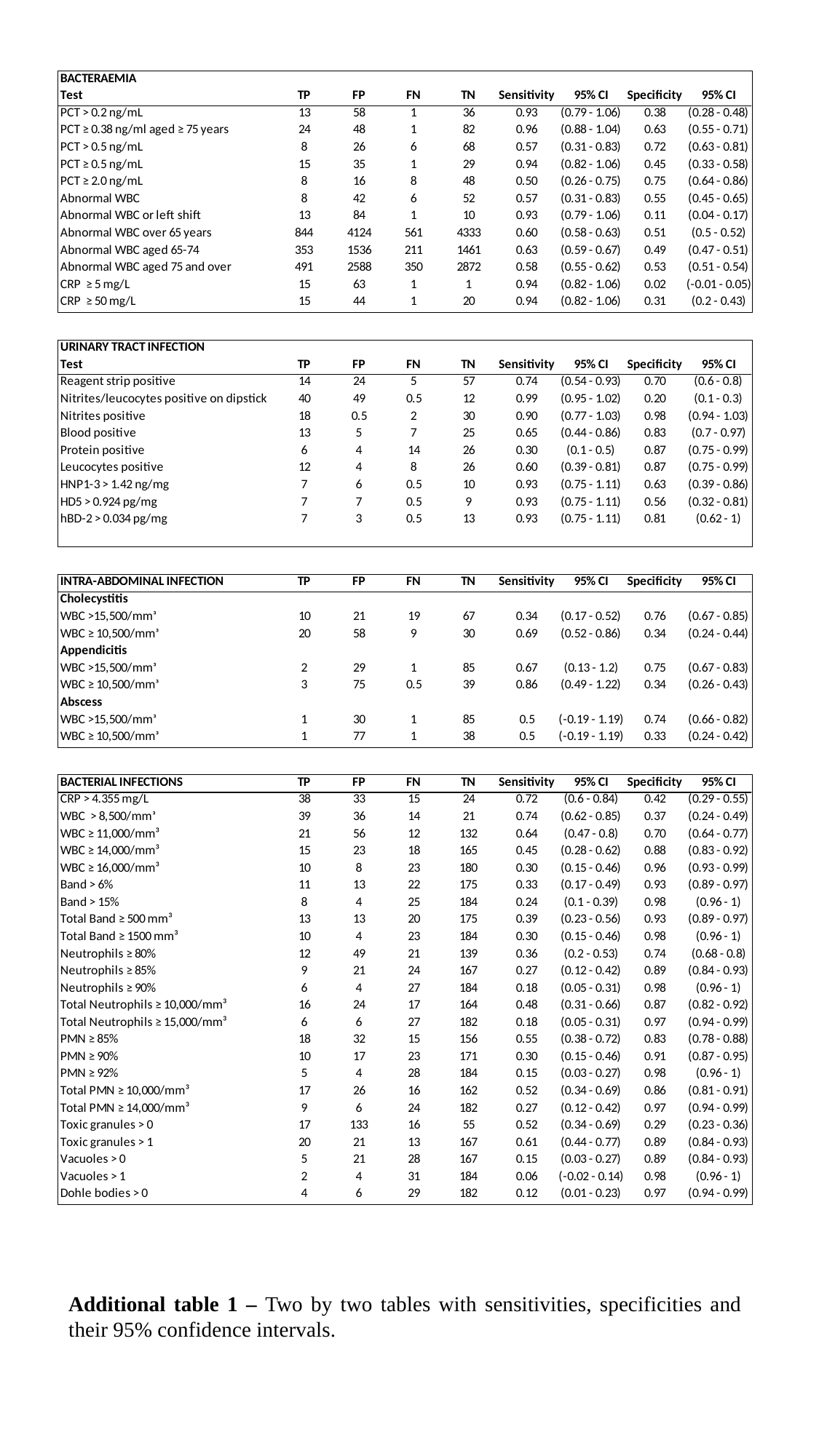

Additional table 1 – Two by two tables with sensitivities, specificities and their 95% confidence intervals.
